# Inhibition of *Clostridioides difficile* Toxins TcdA and TcdB by Ambroxol

**DOI:** 10.3389/fphar.2021.809595

**Published:** 2022-01-04

**Authors:** Sebastian Heber, Lara Barthold, Jan Baier, Panagiotis Papatheodorou, Giorgio Fois, Manfred Frick, Holger Barth, Stephan Fischer

**Affiliations:** ^1^ Institute of Pharmacology and Toxicology, Ulm University Medical Center, Ulm, Germany; ^2^ Institute of General Physiology, Ulm University, Ulm, Germany

**Keywords:** bacterial AB-type protein toxins, ambroxol, *C. difficile* toxins TcdA and TcdB, glucosyltransferase inhibitor, *C. difficile* infections

## Abstract

*Clostridioides (C.) difficile* produces the exotoxins TcdA and TcdB, which are the predominant virulence factors causing *C. difficile* associated disease (CDAD). TcdA and TcdB bind to target cells and are internalized via receptor-mediated endocytosis. Translocation of the toxins’ enzyme subunits from early endosomes into the cytosol depends on acidification of endosomal vesicles, which is a prerequisite for the formation of transmembrane channels. The enzyme subunits of the toxins translocate into the cytosol via these channels where they are released after auto-proteolytic cleavage. Once in the cytosol, both toxins target small GTPases of the Rho/Ras-family and inactivate them by mono-glucosylation. This in turn interferes with actin-dependent processes and ultimately leads to the breakdown of the intestinal epithelial barrier and inflammation. So far, therapeutic approaches to treat CDAD are insufficient, since conventional antibiotic therapy does not target the bacterial protein toxins, which are the causative agents for the clinical symptoms. Thus, directly targeting the exotoxins represents a promising approach for the treatment of CDAD. Lately, it was shown that ambroxol (Ax) prevents acidification of intracellular organelles. Therefore, we investigated the effect of Ax on the cytotoxic activities of TcdA and TcdB. Ax significantly reduced toxin-induced morphological changes as well as the glucosylation of Rac1 upon intoxication with TcdA and TcdB. Most surprisingly, Ax, independent of its effects on endosomal acidification, decreased the toxins’ intracellular enzyme activity, which is mediated by a catalytic glucosyltransferase domain. Considering its undoubted safety profile, Ax might be taken into account as therapeutic option in the context of CDAD.

## Introduction

The gram-positive enterobacterium *Clostridioides (C.) difficile* is the major cause of (nosocomial) hospital-acquired diarrhea and of severe forms of pseudomembranous colitis. Infections with *C. difficile* (CDI) are accountable for up to one-fourth of all cases of antibiotic-associated diarrhea, which has made *C. difficile* an important and emerging enteropathogen (Larson et al., 1978; Bauer et al., 2011). The incidence and the severity of CDIs has increased significantly in recent years, leading to outbreaks of infections in hospitals worldwide (Cartmill et al., 1994). In 2011–2012, the European Center for Disease Control and Prevention assumed that more than 150,000 new CDI cases emerged annually, with an incidence of about 30 cases (per 100,000 population), resulting in more than 8,000 deaths per year (Cassini et al., 2016). Similar rates were also found for the United States of America. Here, CDIs were accountable for more than 220,000 cases among hospitalized patients in 2017, with estimated attributable health care costs of about $1B and more than 12,000 estimated deaths (Lessa et al., 2015; Kordus et al., 2021). All of this led the national public healthcare agency of the United States, the Centers for Disease Control and Prevention, to classify the threat level for *C. difficile* as urgent.

The major virulence factors of *C. difficile* are the two secreted protein toxins A (TcdA) and B (TcdB). The presence of the toxins is sufficient to fully develop the emergence of the characteristic clinical symptoms (Lyras et al., 2009; Kuehne et al., 2010). Both toxins display a high sequence homology and an overall comparable multimodal structure (von Eichel-Streiber et al., 1992). They belong to the group of clostridial glucosyltransferases and are subdivided into at least four distinct domains (Jank and Aktories, 2008; Belyi and Aktories, 2010). The enzymatically active glucosyltransferase domain (GTD) is located at the N-terminal part of the toxins whereas the two middle parts are responsible for toxin processing and translocation into the host cell cytosol. The C-terminal domain mediates the binding of the toxins to their cell surface receptors. Recently, progress has been made in identifying the responsible protein receptors. TcdA and TcdB use different cell surface receptors (Kordus et al., 2021). For TcdA, sulfated glycosaminoglycans and low-density lipoprotein receptor were determined as important host factors responsible for binding and uptake of the toxin (Tao et al., 2019). For TcdB, at least three possible receptors were determined including the Wnt receptor Frizzled (Tao et al., 2016), the chondroitin sulfate proteoglycan 4 (Yuan et al., 2015), and poliovirus receptor-like 3 (LaFrance et al., 2015).

TcdA and TcdB are released from *C. difficile* into the surrounding host tissue where they enter target cells via receptor-mediated endocytosis in a specific toxin-receptor complex (Florin and Thelestam, 1983; Frisch et al., 2003). The acidic milieu in early endosomes leads to conformational changes within the toxins’ structure that enables the insertion of hydrophobic regions inside the translocation domain and subsequent pore formation in endosomal membranes (Henriques et al., 1987; Qa’Dan et al., 2000; Barth et al., 2001). With the help of the transmembrane pores, the GTD translocates from the endosomal lumen to the cytosolic side of the endosomes where an autoproteolytic cleavage, which requires inositol hexakisphosphate (InsP_6_), mediates the release of the GTD into the cytosol (Pfeifer et al., 2003; Reineke et al., 2007). Once in the cytosol, the GTD glucosylates small GTPases of the Rho/Ras-superfamily. TcdA and TcdB covalently transfer a glucose moiety from the co-substrate UDP-glucose to the GTPases, which results in inactivation of signal transduction (Just et al., 1995a; Just et al., 1995b), reorganization of the actin cytoskeleton and cell rounding. The most well defined proteins of the Rho-family are RhoA, Rac1, and Cdc42, all together important key regulators of actin based processes. Both toxins mono-glucosylate Rho proteins by transferring a glucose-residue onto the highly conserved effector domain amino acids threonine 37 in case of RhoA and threonine 35 in case of Rac1 and Cdc42 (Just et al., 1995a). *In vivo*, these actions of the toxins are the reason for gut barrier disruption and the development of the clinical symptoms.

As acidification of endosomal vesicles is essential for the successful translocation of the toxins into the cytosol, we investigated the effects of the licensed muco-lytic drug ambroxol (Ax) on TcdA and TcdB in the context of the present study. Ax contains a lipophilic organic ring system linked to a secondary amine via a short spacer allowing it to cross membranes by diffusion. Ax is a weak base and is predicted to enrich in acidic compartments by protonation where it leads to pH neutralization (Fois et al., 2015). In particular, acidification of endosomal vesicles is essential for the successful translocation of the toxins into the cytosol, which has been demonstrated using bafilomycin A1 (BafA1), an inhibitor of the vacuolar H^+^-ATPase (Barth et al., 2001). Ax protected cells from native TcdA and/or TcdB and, unexpectedly, directly inhibited the glucosyltransferase activity.

## Materials and Methods

### Toxins and Reagents

The native toxins TcdA and TcdB from *C. difficile* VPI 10463 were purified as described earlier (von Eichel-Streiber et al., 1987). *N*-Ethylmaleimide was ordered from Sigma Aldrich, United States. Castanospermine was purchased from Santa Cruz Biotechnology, United States. α-Defensin-5 was ordered from PeptaNova, Germany. Ax was generously provided by Dr. Birgit Jung, Böhringer-Ingelheim Pharma GmbH & Co., KG, Biberach, Germany.

### Cell Culture and Cytotoxicity Assays

Cells were cultured in saturated humidity at 37°C, 5% CO_2_ and reseeded three times per week. Vero cells were cultured using MEM with additions of 10% fetal calf serum (both GIBCO Life Technologies, United States), 1 mM sodium pyruvate, 1 mM L-glutamine, 0.1 mM non-essential amino acids, 100 U/ml penicillin and 100 µg/ml streptomycin. HCT116 cells were cultured under the same conditions using DMEM with 10% fetal calf serum (both GIBCO Life Technologies, United States), 1% sodium pyruvate and 100 U/ml penicillin and 100 µg/ml streptomycin. For imaging, either an Axiovert 40CFL microscope (Zeiss, Germany) with a ProgRes C10 CCD camera (Jenoptik, Germany) or a Leica DMi1 microscope with a Leica MC170 HD camera (both Leica, Germany) was used. Images were processed using ImageJ software (Schneider et al., 2012). For cytotoxicity assays, the respective growth medium of the cells was removed and the toxins in the presence or absence of the single test substances were added to the cells in serum-free medium. Afterwards, the cells were further incubated at 37°C until the respective time points.

### Probing the Intracellular Rac1 Glucosylation Status in Intact Vero Cells After Treatment With TcdB

3 × 10^4^ Vero cells per well were seeded in a 24-well plate 2 days prior intoxication. Intoxication was performed as described in the previous paragraph in serum-free medium. Cells were mechanically harvested using a cell scraper in PBS supplemented with 1× cOmplete^TM^ protease inhibitor (Roche, Germany). After one freeze/thaw cycle, the cell lysate was transferred to SDS-PAGE followed by Rac1 immunoblotting. Mouse anti-non-glucosylated Rac1 antibody (1:500, BD Biosciences, #610650, United States) was used for determination of the glucosylation status, which was normalized to Hsp90 signal (1:1,000, Santa Cruz Biotechnology, #13119, United States).

### Immunofluorescence Microscopy

4 × 10^4^ Vero cells per well were seeded 1 day prior imaging in 8-well µ-slides (ibidi GmbH, Germany). Intoxication was performed as mentioned. Cells were fixed with 4% PFA for 20 min, permeabilized with 0.4% Triton-X100 in PBS for 5 min, treated with 100 mM glycine, 0.1% Tween20 in PBS for 2 min and blocked with blocking buffer (5% skim milk powder, 0.1% Tween®20) for 30 min at 37°C. The cells were washed, immunostained with a specific antibody only recognizing non-glucosylated Rac1 (1:100) in blocking buffer and washed again. For fluorescence analysis, a fluorescently-labeled goat anti-mouse-568 secondary antibody (1:750, Invitrogen, A11004, United States) and phalloidin-FITC (1:100, Sigma Aldrich, P5282, United States) was used for 30 min followed by Hoechst33342 staining (1:5,000, 5 min, both in blocking buffer). iMic Digital Microscope and Live Acquisition 2.6 software (both FEI Munich GmbH, Thermo Fisher Scientific, United States) were used for imaging. Images were processed using ImageJ software.

### Precipitation Studies With TcdB

TcdB stock solution was centrifuged at 14,000 rpm for 20 min at 4°C to remove preformed aggregates. 50 ng of TcdB were incubated in the presence and absence of the respective inhibitors for 30 min at 37°C in a total volume of 35 µl. Aggregated protein was collected as a pellet by centrifugation as mentioned above. 30 µl supernatant were collected and the remaining pellet was resuspended in a total volume of 60 µl PBS. 30 µl of each fraction were subjected for separation to an 8% SDS-polyacrylamide gel and detected by immunoblotting against TcdB. Anti-TcdB-antibody (1:1,000, Abcam, ab270452, United Kingdom) was used for signal detection.

### Analysis of TcdB Binding to Vero Cells

3 × 10^4^ Vero cells per well were seeded in a 24-well plate 2 days prior to analysis. The cells were pre-cooled on ice for 30 min to prevent endocytosis. Ice-cold intoxication medium (serum-free) was used to allow binding of TcdB to the cells for 1 h. After two washing steps with PBS to remove non-bound proteins, cells were harvested by addition of 100 µl pre-heated (95°C) 2.5× Laemmli buffer. Next, cells were scraped off, heated for 10 min at 95°C, and cell lysates were transferred to SDS-PAGE followed by immunoblotting against TcdB (1:1,000, Abcam, ab270452, United Kingdom). Hsp90 was detected as loading control as described above.

### *In Vitro* Glucosylation of Rac1 by TcdB

20 µg total protein from a whole cell lysate (as source for Rac1) in combination with TcdB (10 nM) was used for *in vitro* glucosylation. The reaction was performed in glucosylation buffer (50 mM HEPES, 100 mM KCl, 2 mM MgCl_2_, 1 mM MnCl_2_, 100 mg/L BSA, pH 7.5) for 1 h at 37°C in a total volume of 20 µl. Reaction was stopped by adding 5 µl 5× Laemmli buffer and 10 min heating of the samples at 95°C. The glucosylation status of Rac1 was determined by immunoblotting as described above. Hsp90 was detected as loading control.

### *In Vitro* Enzyme Activity of the Enzyme Component C2I of the Binary *Clostridium (C.) botulinum* C2 Toxin

C2I (1 ng) was supplemented with ADP-ribosylation buffer, whole cell lysate (40 µg) as source for actin, two different concentrations of Ax (100 and 1,000 µM) and 10 µM biotin-labelled NAD^+^ (R&D Systems, #6573/131U, United States) for 30 min at 37°C. Afterwards, reaction was stopped by adding 5× Laemmli buffer and heating at 95°C for 10 min. Then, SDS-PAGE and immunoblotting was performed and biotinylated actin was detected by the enhanced chemiluminescence reaction (ECL) using a peroxidase-coupled streptavidin antibody (Merck, #11089153001, Germany).

### Expression and Purification of Recombinant Rac1

Rac1 was expressed as a recombinant GST-tagged protein in *E. coli* BL21 transformed with the pGEX-4T-2-GST_Rac1 plasmid. Purification was performed as described earlier for other GST-tagged proteins (Barth et al., 1998).

### *In Vitro* Cysteine Protease Activity of TcdB

TcdB (2 µg) was incubated for 1 h at 37°C with 1 mM inositol hexakisphosphate (Santa Cruz Biotechnology, United States) in 20 µl to allow for autoproteolytic cleavage. Reactions were buffered using 20 mM Tris and 150 mM NaCl with pH 7.4 and stopped by addition of 5 µl 5× Laemmli buffer and 10 min heating at 95°C. For analysis, samples were transferred to SDS-PAGE and subsequent Coomassie staining of the gel.

### Hydrolase and Glucosyltransferase Activity of TcdB

UDP-Glo™ Glycosyltransferase Assay with UDP-glucose as cosubstrate (Promega, V6991, United States) was used to monitor hydrolase and glucosyltransferase activity. The assay was performed as described by the manufacturer. In short, reactions were performed for 1 h at 37°C in a total volume of 40 µl of glucosylation buffer. 50 nM (for hydrolase activity) and 200 pM (for glucosyltransferase activity) TcdB was used and for both reactions, 100 µM of UDP-glucose was added. For measuring the glucosyltransferase activity, 5 µM recombinant Rac1 was added as substrate. Thereafter, the preparations were split into three times 10 µl and transferred to a 96-well half-area microplate (Greiner, #675075, Austria). Reactions were stopped by addition of 10 µl UDP Detection Reagent. Content was mixed by shaking at 1,000 rpm for 30 s. Luminescence signal was recorded within 1 h after addition of UDP Detection Reagent using a Tecan infinite M1000Pro plate reader (Tecan Trading AG, Switzerland) with an integration time of 750 ms.

### Statistics

All experiments were performed at least three times as independent replicates. Each replicate was carried out at minimum in duplicate. For statistical analysis, ordinary one-way ANOVA was performed with Dunnett’s multiple comparison test (GraphPad, Version 6). Resulting *p* values were indicated as follows: ns, not significant; *p < 0.05; **p < 0.01.

## Results

### Ax Protects Vero and HCT116 Cells From *C. difficile* TcdA and TcdB

**FIGURE 1:**
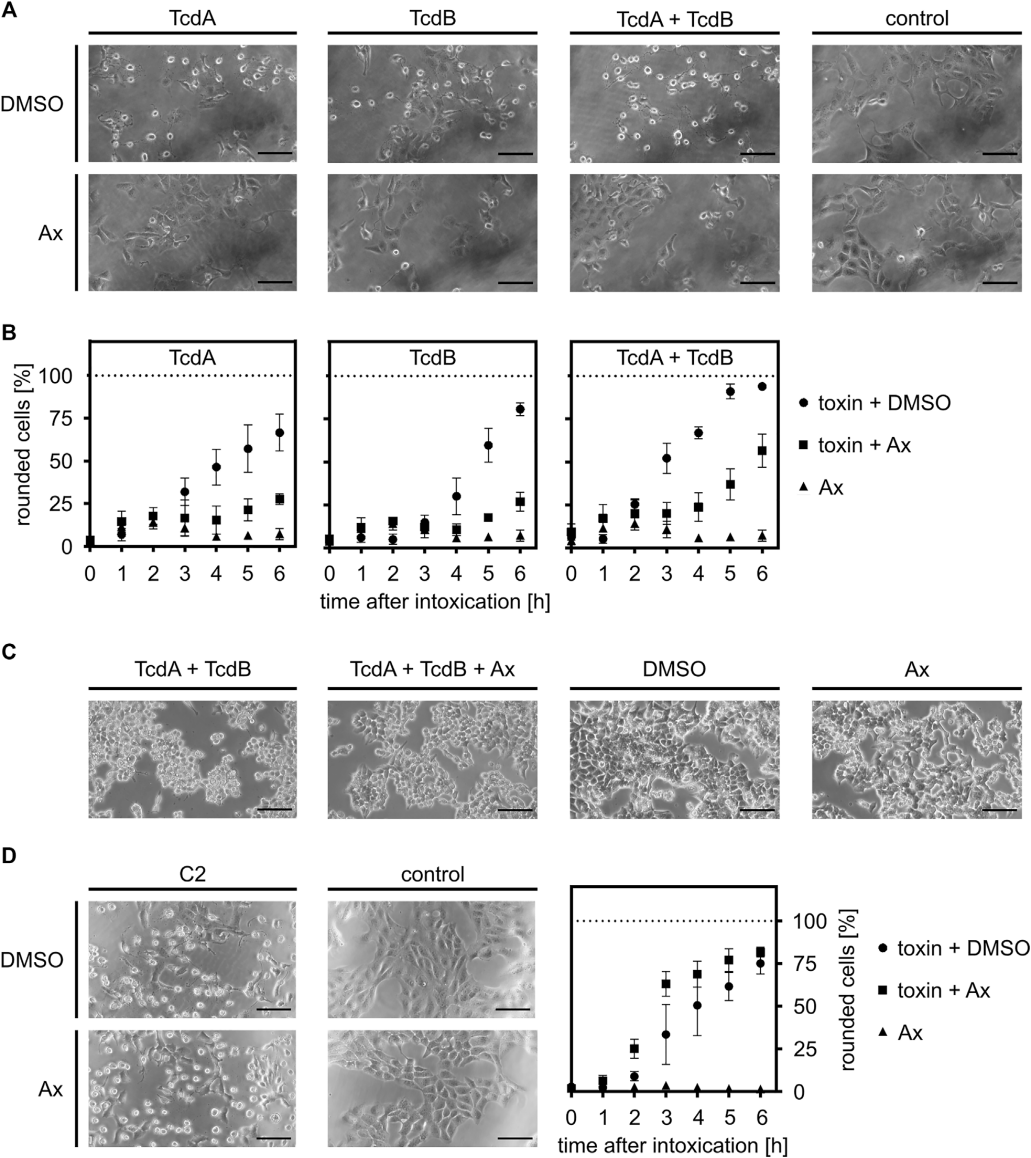
Ax protects cells from intoxication with TcdA, TcdB and the combination of both toxins. **(A)** Vero cells were intoxicated with 10 pM TcdA, 10 pM TcdB or the combination of both (each 10 pM) in the presence or absence of Ax (150 µM) or DMSO as solvent control. Representative pictures after 5 h are depicted (*n* = 3). **(B)** At indicated time points, pictures were taken and the amount of rounded Vero cells was determined. Depicted is the ratio of rounded cells compared to total cell number. Values are given as mean ± standard deviation (SD) of three technical replicates. Biological replicates showed comparable inhibitions of intoxication (*n* = 3). **(C)** HCT116 cells were treated as in **(A)**. 100 pM of the toxins were used. Images depict representative cells after 4 h incubation. **(D)** Vero cells were incubated with C2 toxin (C2I: 1 nM, C2IIa: 1.66 nM) in the presence or absence of Ax (150 µM) or DMSO as solvent control. Pictures were taken 5 h after intoxication. Intoxication kinetics are depicted as mentioned for **(B)**. Scale bars correspond to 100 µm.

In the first set of experiments, the effect of Ax on intoxication of cells with TcdA or TcdB was investigated in the well-established cell-rounding assay with Vero cells. These cells are very sensitive towards both toxins and display a clear and robust response in terms of rounding (Figure 1A). When applied concomitantly, i.e., without any pre-incubation period, the number of round cells challenged with either TcdA or TcdB and Ax (150 µM) was significantly lowered compared to cells treated only with the toxins, indicating that Ax reduces the intoxication of the cells. Moreover, Ax protected cells from the intoxication by the medically relevant combination of TcdA and TcdB (Figure 1A). The quantitative analysis of the toxin-induced changes in cell morphology over time revealed protection of cells by Ax even after 6 h (Figure 1B). This result was morphologically confirmed in the physiologically and medically more relevant human colon cancer cell line HCT116 (Figure 1C). For both cell lines, Ax alone did not cause any substantial changes in cell morphology (Figures 1A, C) or cell viability (Supplementary Figure S1). Interestingly, the binary actin ADP-ribosylating C2 toxin from *C. botulinum* was not affected by Ax (Figure 1D), demonstrating a selective mode of action of Ax against TcdA and TcdB. Moreover, since C2 toxin also requires acidic endosomes for its cellular uptake, this result suggests another mechanism underlying the inhibitory effect of Ax towards TcdA/TcdB. Even at comparatively high concentrations of TcdB, a significant and clear reduction in TcdB-induced cell rounding was observed (Supplementary Figure S2). Because TcdB was more cytotoxic than TcdA in earlier studies (Just and Gerhard, 2004) and considered as the major virulence factor of *C. difficile* (Carter et al., 2015), all further experiments investigating the underlying molecular mechanism were performed with TcdB.

### Less Rac1 is Glucosylated in Intact Cells by TcdB in the Presence of Ax

**FIGURE 2:**
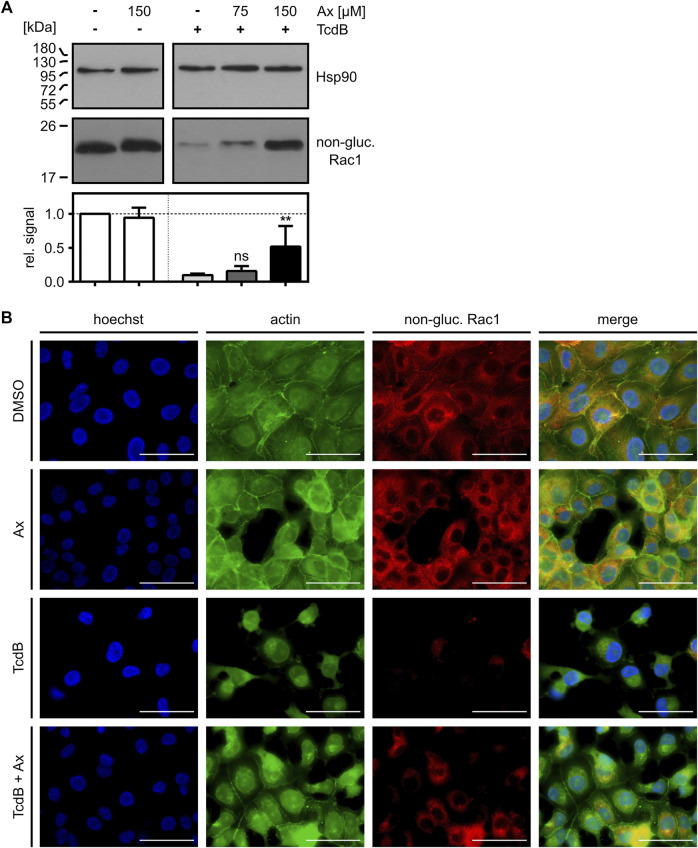
Ax prevents TcdB-induced intracellular Rac1 glucosylation. **(A)** Vero cells were intoxicated with TcdB (10 pM) in the presence or absence of increasing concentrations of Ax or DMSO as solvent control. After 5 h, cells were harvested, lysed and transferred to Western blotting. Non-glucosylated Rac1 signal was normalized to Hsp90 loading control. Relative signal intensities are given as mean ± SD (*n* = 4). Ordinary one-way ANOVA was performed with Dunnett’s multiple comparison test against toxin-only control. Asterisks indicate significance levels for toxin containing samples, with ns (not significant), *p < 0.05 and **p < 0.01. **(B)** Vero cells were treated with TcdB (10 pM), Ax (150 µM) and DMSO as solvent control. After 5 h, cells were fixated and immunofluorescence staining was performed. Nuclei (blue), actin cytoskeleton (green) and non-glucosylated Rac1 (red) were stained. Representative images of the individual channels and the merge of all three are depicted. Scale bars correspond to 50 µm.

To further elucidate the protective effect of increasing concentrations of Ax against TcdB, the glucosylation status of intracellular Rac1 was analyzed in more detail by immunoblotting with an antibody that specifically recognizes non-glucosylated Rac1 (Genth et al., 2006; Egerer et al., 2007; Fischer et al., 2020; Korbmacher et al., 2020). This experiment revealed that less Rac1 was glucosylated in intact cells in the presence of 150 µM of Ax after incubation with TcdB (Figure 2A). The results were confirmed by an alternative, immunofluorescence microscopy-based approach, where the glucosylation status of Rac1 was analyzed with the same antibody in cells displaying their native morphology (Figure 2B). Treatment of Vero cells with TcdB resulted in an almost complete glucosylation of intracellular Rac1, as indicated by the virtually entire disappearance of the non-glucosylated Rac1 signal. Also, the F-actin structure is highly impaired, as illustrated by the typical rounding of the cells. In the presence of Ax, non-glucosylated Rac1 as well as F-actin were more comparable to the untreated control cells.

### Investigation of the Underlying Molecular Mechanism of TcdB-Neutralization by Ax

**FIGURE 3:**
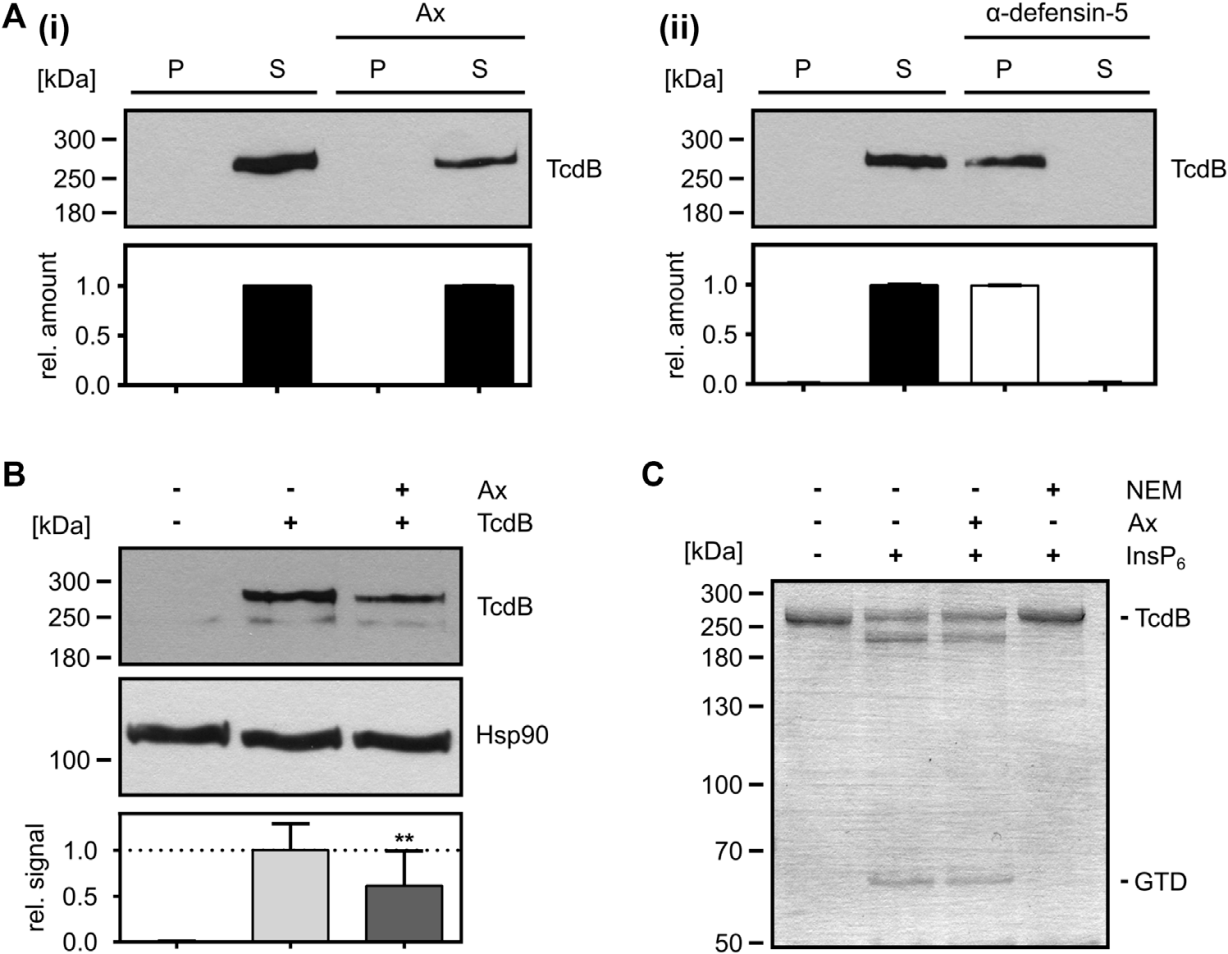
Mode-of-inhibition of Ax as inhibitor of TcdB. **(A)** TcdB (50 ng) was incubated in the presence or absence of Ax (1 mM, **i**) and α-defensin-5 (6 µM, **ii**) for 30 min at 37°C. Samples were centrifuged, separated into supernatant (S) and pellet (P) fraction and analyzed by Western blotting. Relative signals within the two fractions are compared as relative amount of the sample (S + P). Relative amounts are given as mean ± SD (*n* = 3). **(B)** Precooled Vero cells were treated with TcdB (500 pM) to allow for toxin binding. Binding of TcdB was analyzed in the presence of Ax (150 µM) or DMSO as solvent control. Cells were washed, harvested and bound TcdB was analyzed by Western blotting. Relative signal intensities are given as mean ± SD (*n* = 3). A representative Western blot is depicted. Ordinary one-way ANOVA was performed with Dunnett’s multiple comparison test against toxin-only control. Asterisks indicate significance levels for toxin containing samples, with ns (not significant), *p < 0.05 and **p < 0.01. **(C)** *In vitro* cysteine protease activity of TcdB (2 µg) was analyzed in the presence of InsP_6_ (1 mM) to induce cysteine-protease activity. Ax (1 mM) or NEM (1 mM) were added. Cysteine protease activity was analyzed after 1 h at 37°C by SDS-PAGE and subsequent Coomassie staining. After successful cleavage, GTD (∼63 kDa) is released from full length TcdB (∼270 kDa). One representative SDS-PAGE is depicted.

From the observation that less Rac1 was glucosylated by TcdB in intact cells in the presence of Ax, it cannot be distinguished whether Ax directly inhibits the enzyme activity of the GTD or prevents the transport of the GTD into the host cell cytosol, or both. Therefore, we investigated the effect of Ax on the individual steps of TcdB uptake into cells. First, the capability of Ax to directly precipitate and thereby sequester TcdB was investigated. To this end, TcdB was incubated with or without Ax and centrifuged to obtain potential toxin-aggregates as described earlier (Korbmacher et al., 2020). As depicted in Figure 3A, TcdB was present in the supernatant fraction after incubation with Ax (i), indicating that Ax does not form insoluble aggregates with TcdB. In contrast, incubation of TcdB with α-defensin-5, a peptide for which we demonstrated earlier that it precipitates TcdB (Korbmacher et al., 2020), resulted in an almost quantitative TcdB precipitation demonstrating that this assay works under the chosen conditions (ii). Next, the influence of Ax on the binding of TcdB to cultured cells was investigated. Therefore, cells were cooled down to 4°C to reduce endocytosis to a minimum and incubated with TcdB in the presence and absence of Ax. Subsequently, cells were washed and bound TcdB was analyzed via immunoblotting. Here, the amount of cell-bound TcdB was slightly reduced in the presence of Ax (Figure 3B). However, this modest reduction might not fully explain the strong inhibition of TcdB intoxication of cells by Ax. Therefore, further steps of toxin uptake were examined, such as the intracellular processing of TcdB. It was tested whether Ax has an effect on the cysteine protease domain (CPD) of TcdB *in vitro* by analyzing the intrinsic autoproteolysis of the toxin in the presence of InsP_6_. As a result, it was clearly visible that Ax did not affect InsP_6_-induced autocatalytic processing of TcdB indicating that the CPD-related activity of TcdB is not affected by Ax (Figure 3C). Noteworthy, *N*-ethylmaleimide (NEM), an established CPD inhibitor (Egerer et al., 2007), prevented the autocatalytic processing of TcdB in the same experiment.

### Effect of Ax on the GTD of TcdB *In Vitro*

**FIGURE 4:**
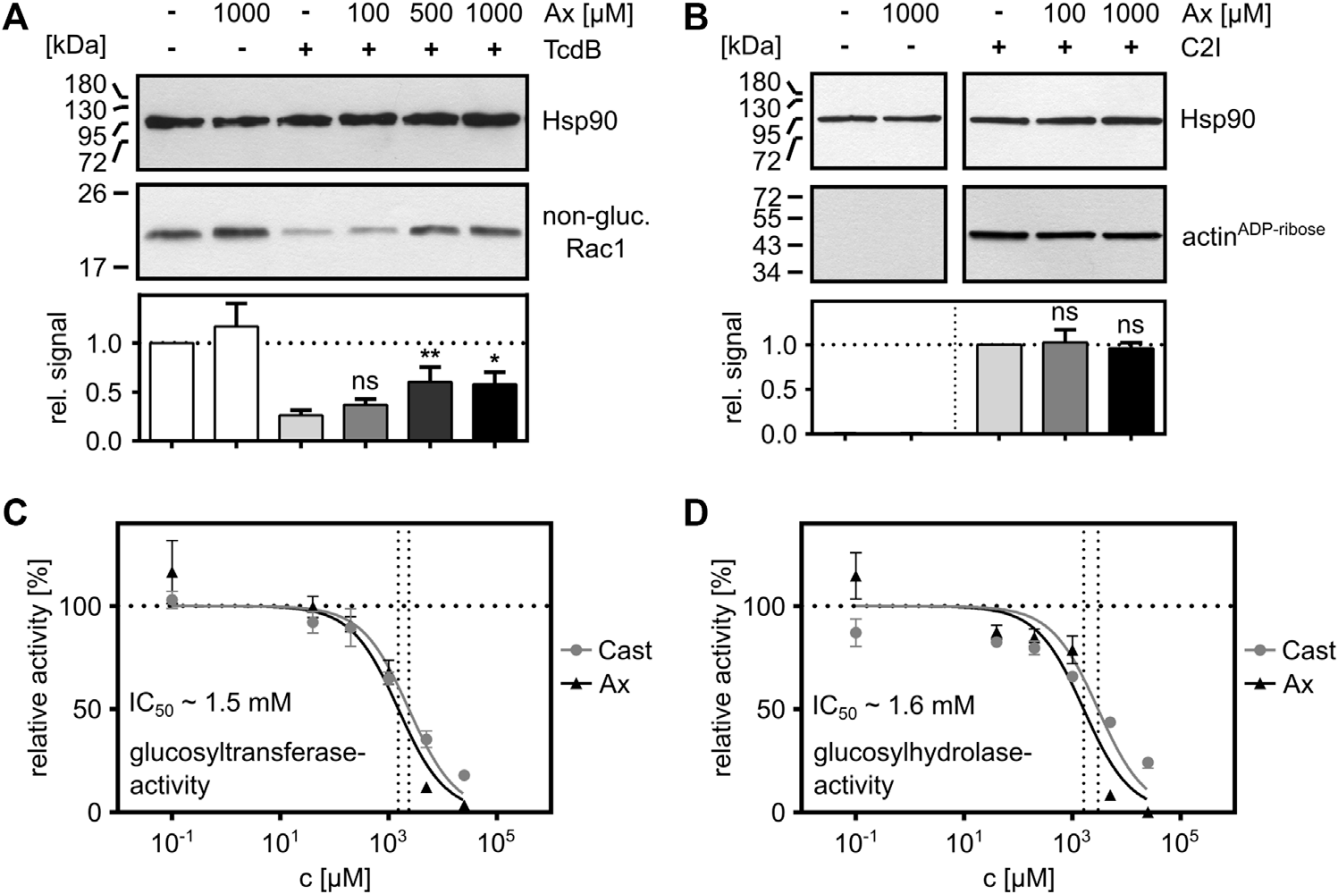
Ax inhibits the enzyme activity of TcdB *in vitro*. **(A)** Whole cell lysate (20 µg) as source for Rac1 was supplemented with TcdB (10 nM) in the presence or absence of Ax (150 µM) or DMSO (solvent control) for 2 h at 37°C. Afterwards, samples were subjected to SDS-PAGE followed by Western blotting. Non-glucosylated Rac1 signals are normalized to Hsp90 loading control. Relative signals to toxin control are given as mean ± SD (*n* = 4). A representative Western blot image is depicted. Ordinary one-way ANOVA was performed with Dunnett’s multiple comparison test against toxin-only control. Asterisks indicate significance levels for toxin containing samples, with ns (not significant), *p < 0.05 and **p < 0.01. **(B)** C2I (1 ng) was incubated with whole cell lysate (40 µg) with two different concentrations of Ax (100 µM, 1,000 µM) and DMSO as solvent control for 30 min at 37°C. ADP-ribosylated and thereby biotin-labeled actin (actin^ADP-ribose^) signals are normalized to Hsp90 loading control. Relative signals to toxin control are given as mean ± SD (*n* = 3). A representative Western blot is depicted. Ordinary one-way ANOVA was performed with Dunnett’s multiple comparison test against C2I-only control. Asterisks indicate significance levels for toxin containing samples, with ns (not significant). **(C)** TcdB (200 pM) and recombinant Rac1 (5 µM) were incubated with increasing concentrations of Ax and castanospermine (Cast) to analyze glucosyltransferase activity of TcdB by UDP-Glo™ glycosyltransferase assay. Nonlinear fit was applied with Graphpad Prism via log(inhibitor) vs. normalized response function. The estimated IC_50_ value for Ax (∼1.5 mM) is displayed in the graph. **(D)** TcdB (50 nM) was incubated with increasing concentrations of Ax and castanospermine (Cast) to analyze glucosylhydrolase activity of TcdB by UDP-Glo™ glycosyltransferase assay. Nonlinear fit was applied with Graphpad Prism via log(inhibitor) vs. normalized response function. The estimated IC_50_ value for Ax (∼1.6 mM) is displayed in the graph.

Finally, the effects of Ax on the intracellular glucosylation activity of TcdB were investigated in more detail. For this purpose, the glucosylation of Rac1 by TcdB was analyzed *in vitro* by incubating TcdB with whole cell lysate (as a source for Rac1) in the presence and absence of increasing concentrations of Ax. Here, a clear concentration-dependent inhibition of the enzyme activity of TcdB by Ax was observed (Figure 4A). As expected from our earlier result that C2 toxin is not affected by Ax in the cell rounding assay (Figure 1D), no effect by Ax was detected on the enzyme activity of the ADP-ribosyltransferase C2I (Figure 4B), suggesting that Ax is a specific inhibitor of glucosyltransferases such as TcdA and TcdB. To get further insights into the underlying inhibitory mode of action of Ax, the glucosyltransferase and glucosylhydrolase activities of TcdB were analyzed. Ax was not only able to reduce the glucosyltransferase activity (Figure 4C) but also the glucosylhydrolase activity (Figure 4D). For both activities, similar IC_50_ values were determined for Ax and compared with castanospermine, a well-established glucosidase inhibitor (Jank et al., 2008). Noteworthy, the addition of Ax to either the glucosylation buffer or the culture medium did not result in any changes in the respective pH values (Supplementary Figure S3).

## Discussion

Infections with the human-pathogenic bacterium *C. difficile* persist to be a major challenge for healthcare systems in Western countries. CDI come along with a wide range of gastrointestinal diseases characteristically in hospitalized patients treated with broad-spectrum antibiotics, which reduces the abundance of the protective host microbiota in the gastrointestinal tract (Theriot et al., 2016). Although there are therapeutic options with some specific antibiotics such as vancomycin, fidaxomicin or metronidazole against CDI (Louie et al., 2011; Tart, 2013), pharmacological inhibitors against the produced toxins, which are the major virulence factors in this context, are urgently needed in addition to antibacterial drugs to neutralize the toxins TcdA and TcdB. In this study, the commonly used muco-lytic drug Ax was identified as a potent inhibitor of TcdA and TcdB, as well as their physiologically more relevant combination, in cell models.

Ax has mucociliary as well as mucokinetic effects and is used worldwide to treat acute and chronic respiratory diseases (Gupta, 2010). Recent studies revealed therapeutic effects of Ax also against Parkinson’s disease (McNeill et al., 2014) and against various viruses like rhinovirus (Yamaya et al., 2014) and SARS-CoV-2 (Carpinteiro et al., 2021), in part due to the ability of Ax to accumulate in acidic vesicles such as late endosomes and lysosomes, where it neutralizes intravesicular pH levels. This property makes Ax an attractive candidate to examine its inhibitory potential against bacterial toxins that essentially require the acidification of endosomes for their uptake into human cells.

Many bacterial protein toxins rely on acidification of early endosomes to translocate from endosomal vesicles into the cytosol of their target cells. Prominent examples are anthrax toxin (Ménard et al., 1996; Young and Collier, 2007), *C. botulinum* C2 toxin (Barth et al., 2000) or diphtheria toxin (Madshus et al., 1991), but also the toxins TcdA and TcdB from *C. difficile* (Aktories et al., 2017). Given that inhibition of vacuolar H^+^-ATPase with BafA1 reliably inhibits intoxication of cells by all bacterial toxins that exploit acidic endosomes (Umata et al., 1990; Barth et al., 2001; Gerhard et al., 2013), we initially challenged cells with the native *C. difficile* toxins TcdA and/or TcdB, or with C2 toxin in the presence and absence of Ax.

Intoxication of eukaryotic cells by those toxins is characterized by high specificity and efficiency and a clear change in cell morphology (rounding up). Thus, analysis of cell rounding represents an ideal endpoint to monitor intoxication processes. It was surprising that Ax was capable of inhibiting TcdA, TcdB and their combination, whereas C2 toxin was not affected. This fact argued against a universal inhibitory mechanism of Ax against bacterial toxins that are internalized via acidic endosomes. However, the role of Ax-induced neutralization of acidic endosomal pH could not be fully elucidated, and why the intoxication of cells by C2 toxin is not inhibited by Ax. One possible explanation might be that the pH of the endosomes is still acidic enough for the C2 toxin to deliver its enzyme subunit C2I into the cytosol. For C2 toxin, a pH value below pH 5.5 is described to be sufficient for successful translocation of the enzyme component C2I into the cytosol (Blöcker et al., 2003). The endosomal pH for successful translocation of TcdB however is described to be below pH 4 (Lanis et al., 2010). Yet this still needs to be clarified in the future.

In the present study, the detailed effect of Ax on clostridial glucosylating toxins was investigated for TcdB. The time- and concentration-dependent reduction of TcdB-cytotoxicity by Ax was confirmed using different methods relying on changes in cell morphology and intracellular substrate modification (immunoblot analyses and fluorescence microscopy). Internalization of TcdB is a multi-modal process. To unravel the underlying molecular mode of inhibition, individual steps during the intoxication process were evaluated more extensively. First of all, it was examined whether Ax is able to form biologically inactive aggregates with TcdB, similar to what has been observed with α-defensins, which also act as bacterial toxin-inhibitors (Giesemann et al., 2008; Korbmacher et al., 2020). Since there was no obvious aggregation of TcdB, binding of TcdB to the cell surface was studied. In our opinion, the reduction observed here was not sufficient to fully explain the strong inhibition of TcdB by Ax, so the following step was to analyze the toxins’ intramolecular autoprotease activity. In general, not the full-length toxin but only the GTD reaches the cytosol of target cells (Pfeifer et al., 2003). After translocation across the endosomal membrane, TcdB is autoproteolytically cleaved in the presence of intracellular InsP_6_ (Reineke et al., 2007). However, we could exclude an effect of Ax on this step, which occurs immediately before substrate modification. Most interestingly, Ax had a marked effect on the glucosylation levels of Rac1 when treated with TcdB. In the presence of Ax, a clear inhibition of the glucosyltransferase activity with IC_50_ values in the low millimolar range was obtained. At comparable concentrations, Ax also inhibited glucosylhydrolase activity of TcdB in the absence of its natural substrate Rac1, indicating neither an influence on the GTD-Rac1 interaction nor a direct interaction between Ax and Rac1. In our hands, Ax was as potent as castanospermine, an already known inhibitor of the enzyme domain of TcdB (Jank et al., 2008). Hydrolase activities are known for several bacterial toxins, such as for various ADP-ribosyltransferases. In this case, the enzymatically active ADP-ribosyltransferases catalyze the attachment of an ADP-ribose residue to specific target proteins of the host through glycosidic bonds. However, in the absence of the target substrate, ADP-ribosyltransferases possess NAD glycohydrolase activity, resulting in the cleavage of intracellular NAD into ADP-ribose and nicotinamide (Deng and Barbieri, 2008). For TcdA and TcdB, similar activities were found (Ciesla and Bobak, 1998). Both toxins are able to cleave intracellular UDP-glucose (UDP-Glc) into glucose and UDP. In the presence of their target substrates, the toxins glucosylate GTPases at a key threonine. In the absence of an appropriate acceptor protein, though, they hydrolyze the nucleotide-sugar UDP-Glc to UDP and free glucose (Bhattacharyya et al., 2002; Reinert et al., 2005).

Ax is a small molecule that acts as a radical scavenger and that is composed of a primary aromatic and a secondary amine. It has been used in medical applications for almost four decades and underwent a broad range of toxicity studies revealing a low toxicity and a favorable safety profile (Cazan et al., 2018). Typically, Ax is administered in different pharmaceutic formulations with an absolute bioavailability of about 79% (Malerba and Ragnoli, 2008). Depending on the formulation and the doses applied, peak plasma concentrations of Ax greater than 150 ng/ml (around 360 µM) can be achieved (Rojpibulstit et al., 2003). Thus, concentrations used in this study are plausible and Ax alone did not show any adverse side effects on different mammalian cell lines. Also for TcdB, concentrations are below those reported in literature. For mild forms of CDI, stool TcdB-concentrations around 1.3 ng/ml (∼5 pM) were reported, whereas for severe forms of CDI, stool toxin concentration achieved levels up to 111 ng/ml (∼410 pM) (Ryder et al., 2010). Nevertheless, in our attempts, a TcdB concentration up to 500 pM was inhibited by Ax.

Taken together, we identified the licensed drug Ax as a novel potent inhibitor of the clinically important toxins TcdA and TcdB in living cells and *in vitro*. Prompted by the results from this study, it will be interesting to investigate whether Ax also inhibits these toxins *in vivo* and whether Ax is able to neutralize further bacterial toxins of the glucosyltransferase family.

## Data Availability

The original contributions presented in the study are included in the article/Supplementary Material, further inquiries can be directed to the corresponding authors.
