# Supplementary material for: Inhibition of Clostridioides difficile Toxins TcdA and TcdB by Ambroxol
Source: Front Pharmacol. 2022 Jan 4;12:809595. doi: 10.3389/fphar.2021.809595 (PMC8764291; doi:10.3389/fphar.2021.809595)
Supplement: Supplementary file 1 [file DataSheet1.docx]

Supplementary Material

# Supplementary Figures

## Supplementary Figure 1


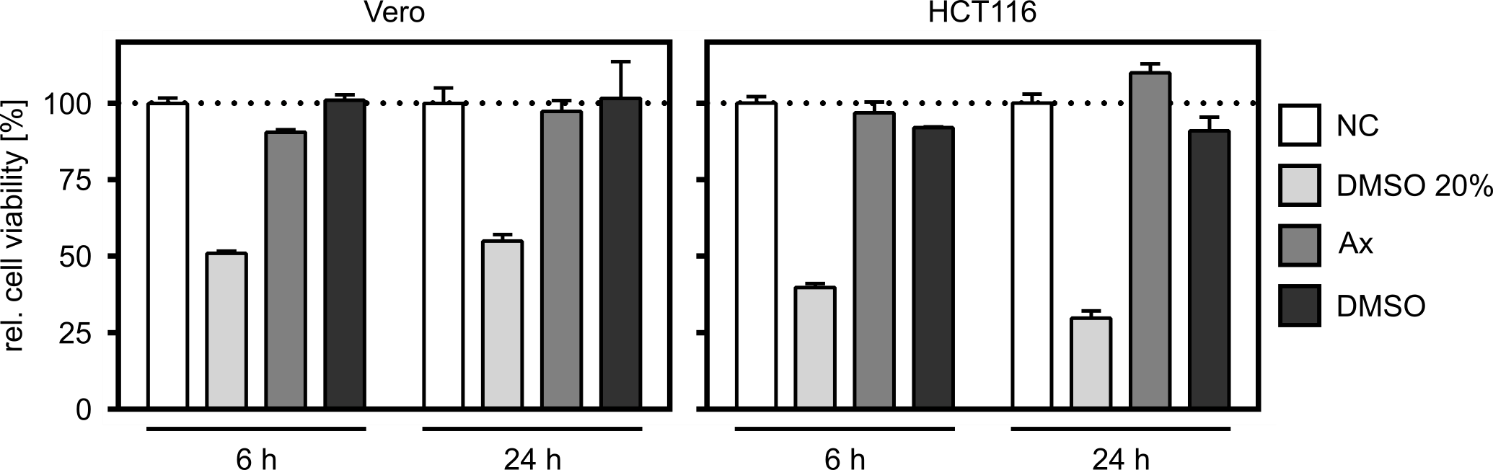


**The applied concentration of Ax has no adverse effect on viability of either Vero or HCT116 cells.** Vero or HCT116 cells were treated with or without Ax (150 µM) for 6 and 24 h. DMSO 20% (light grey bar) was used as positive control for cytotoxicity, whereas DMSO (black bar) represents solvent control. After indicated incubation times, a cell viability assay (CellTiter 96® AQ_ueous_ One Solution Cell Proliferation Assay (MTS), Promega) was performed according to the manufacturer’s instruction.

## Supplementary Figure 2


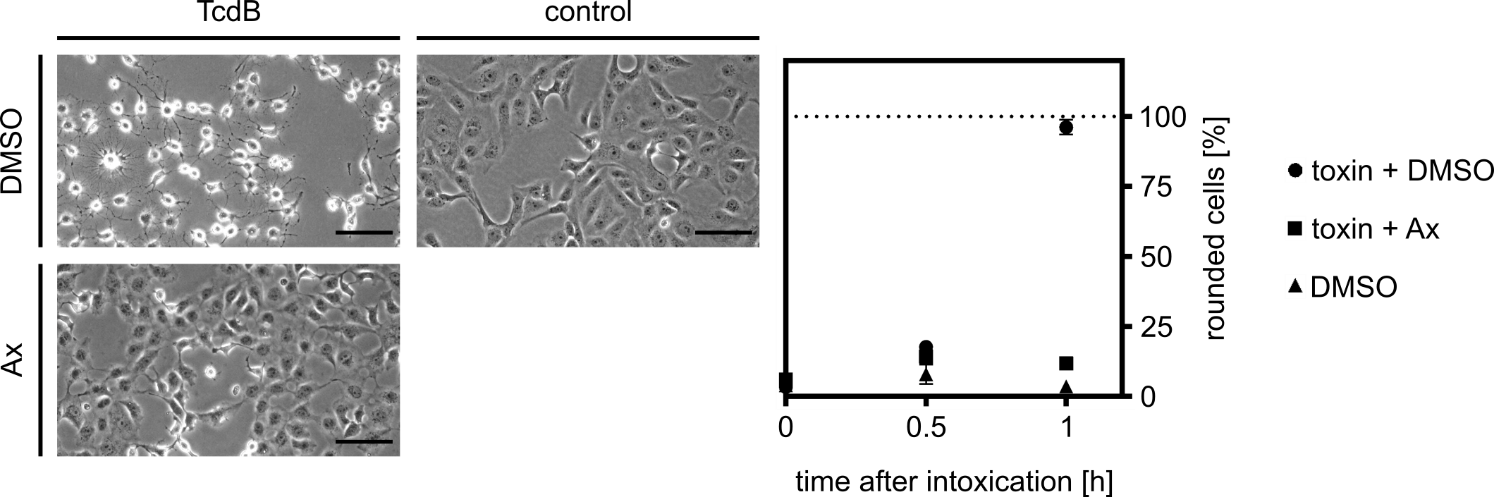


**Ax also protects Vero cells from intoxication with high concentration of TcdB.** Vero cells were treated with TcdB (500 pM) in the presence and absence of Ax (150 µM). DMSO was used as solvent control. Representative images after 1 h are depicted. Scale bars correspond to 100 µm. Ratio of the rounded cells compared to total cell numbers are shown. Values are given as mean ± SD (n = 3).

## Supplementary Figure 3


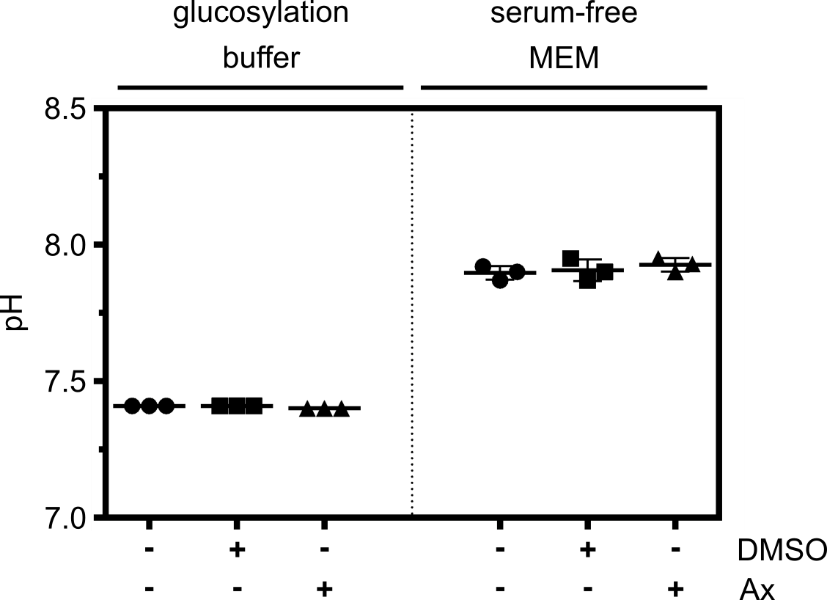


**Ax has no influence on the pH under experimental conditions.** Glucosylation buffer and serum-free MEM were supplemented with Ax or the corresponding volume of DMSO as solvent control. Glucosylation buffer was supplemented with 1 mM Ax and serum-free MEM with 150 µM Ax. Samples were mixed well and the pH was measured in three independent preparations. Mean pH ± SD are given (n = 3).
